# Communication between Bacteria and Their Hosts

**DOI:** 10.1155/2013/361073

**Published:** 2013-12-08

**Authors:** Primrose Freestone

**Affiliations:** Department of Infection, Immunity and Inflammation, University of Leicester, Maurice Shock Medical Sciences Building, University Road, Leicester LE1 9HN, UK

## Abstract

It is clear that a dialogue is occurring between microbes and their hosts and that chemical signals are the language of this interkingdom communication. Microbial endocrinology shows that, through their long coexistence with animals and plants, microorganisms have evolved sensors for detecting eukaryotic hormones, which the microbe uses to determine that they are within proximity of a suitable host and to optimally time the expression of genes needed for host colonisation. It has also been shown that some prokaryotic chemical communication signals are recognized by eukaryotes. Deciphering what is being said during the cross-talk between microbe and host is therefore important, as it could lead to new strategies for preventing or treating bacterial infections.

## 1. Introduction: Bidirectional Communication between Bacteria and Their Hosts

Although bacterial growth and virulence are known to be influenced by local environmental parameters such as temperature, pH, and nutrient availability, the influence of host signals on bacterial behaviour has only recently become apparent. Microbial endocrinology is a newly recognised microbiology research area that has as its foundation the tenet that through their long coexistence with animals and plants, microorganisms have evolved systems for sensing host-associated chemicals such as hormones. These hormone sensors enable the microbe to recognise that they are within the locality of a suitable host and, for commensals, that it is the appropriate time to initiate expression of genes involved in host colonisation or in the case of pathogens, genes for virulence determinants [1–8]. To date, the majority of microbial endocrinology investigations have focused on the interaction of bacteria with stress-associated biochemicals, such as the catecholamine fight and flight hormones adrenaline, noradrenaline, and dopamine [1–7]. This came about because of the long-held view that stress in humans and animals increases their risk of developing an infection due to stress hormone reductions in immune function [9]. However, viewing host stress through the lens of microbial endocrinology provides a broader appreciation of what may be happening with our billions of microbial inhabitants by also considering the impact of the stress event from the perspective of the microbe. In the context of animal welfare, microbial endocrinology has been shown to provide a useful platform on which to develop a holistic understanding of the factors that shape the interactions between microbes and their host during health and disease [1–8].

Although this review will focus on host-microbe communication, it is important to appreciate that a variety of languages are spoken within the kingdoms of life and that evidence will be presented that the chemical signals released by prokaryotes also convey important information to their eukaryotic host. Consideration will therefore also be given to the importance of bacterial quorum sensing signals to the infectious disease process.

## 2. Microbial Endocrinology: Host-Bacteria Communication

Reports dating back over 80 years have found an association between stress hormones and microbial infection, most of which had been assumed to be due to the catecholamines suppressing immune function [9]. For instance, in 1930 Renaud and Miget [10] reported a case of gas gangrene that developed at the injection site of a human patient only six hours after an injection of adrenaline for urticaria. It was later found that the glass syringe used to inject the catecholamine had been sterilised prior to use with alcohol but was still contaminated with the spores of* Clostridium perfringens*. A report from Cooper showed similar findings [11], leading Evans et al. in 1948 [12] to investigate the direct role of catecholamines in the establishment of a bacterial infection. Cultures were mixed with either adrenaline or a saline control and then coinjected into guinea pigs whose tissues were then examined for bacterial growth. Coadministration of the catecholamine produced significantly more bacterial proliferation in the test animals than did the saline. However, because it was not possible to determine the underlying mechanism of this growth enhancement, it was suggested that adrenaline was either forming a protective coat around the bacteria or else had inhibited immune function.

Clear proof that catecholamine stress hormones were recognised by bacteria did not occur until 1992, when Lyte and Ernst used a serum-based test medium to show that noradrenaline and adrenaline catalysed several log-fold increases in growth of *Escherichia coli*, *Yersinia enterocolitica*, and *Pseudomonas aeruginosa* [13]. Seven years later the examination of a larger set of 23 clinical isolates showed that catecholamine recognition was widespread amongst Gram-negative and Gram-positive bacteria [14]. Published reports of how catecholamine stress hormones can modulate bacterial growth and virulence since these initial studies are now many, and a current list of stress-hormone responsive bacteria is shown in Table 1. Shortly after the pioneering study by Lyte and Ernst [13], Lyte coined the term “microbial endocrinology” to describe the phenomenon of microbes recognising eukaryotic hormones [1] which is now the term given to this research field.

## 3. Catecholamine Hormone Responsive Bacteria

**Figure 1:**
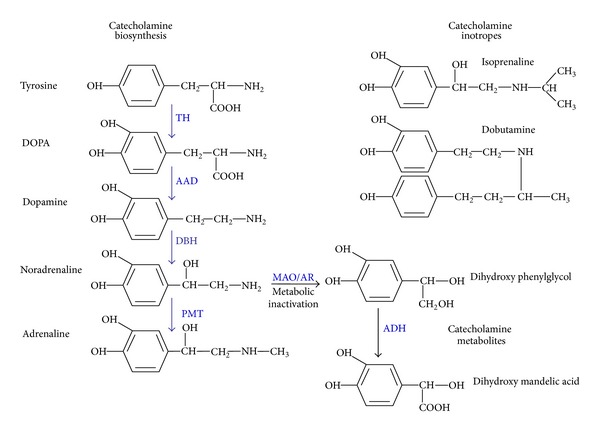
Structures of catecholamine stress hormones, inotropes, and their metabolites. The figure shows the pathway of catecholamine synthesis; it should be noted that for clarity the various cofactors utilised in the pathway are not shown. In mammals, dopamine is synthesized from L-dopa, obtained from dietary sources (such as the amino acids tyrosine and phenylalanine) [15]. Synthesis of catecholamines is to a degree tissue specific; for example, phenylethanolamine N-methyltransferase, which is required for adrenaline synthesis, is not expressed in cells of the enteric nervous system [15, 16]. Catecholamine biosynthesis, TH: tyrosine hydroxylase, AAD: aromatic L-amino decarboxylase, DBH: dopamine *β*-hydroxylase, PMT: phenylethanolamine N-methyltransferase. Metabolic inactivation: MAO: monoamine oxidase, AR: aldehyde reductase, ADH: aldehyde dehydrogenase. This figure was adapted with permission from Freestone et al. (2008) [3].

Catecholamine hormones are a group of widely acting effector compounds derived from tyrosine and other dietary sources, structurally comprising a benzene ring with two adjacent hydroxyl groups and an opposing amine side chain [15]. The synthesis pathway for catecholamine stress hormones begins with L-dopa (mostly derived from dietary sources) which is enzymatically converted → dopamine → noradrenaline → adrenaline (Figure 1). As well as having endocrinological roles, dopamine, noradrenaline, and adrenaline function as neurotransmitters. Noradrenergic and dopaminergic nerve terminals are widely distributed throughout the mammalian body, including the gastrointestinal tract where they make up part of the enteric nervous system (ENS) [15, 16]. Within the gut, noradrenaline is released from storage within sympathetic nerve fibres within the prevertebral ganglia that innervate the gut mucosa, and it has been shown that up to half of the noradrenaline made within the mammalian body is synthesised and utilised within the ENS. Dopamine is produced in nonsympathetic enteric neurons located within the intestinal wall [15, 16]. The abundance of noradrenaline and dopamine containing nerve terminals in the ENS makes the gut a noradrenaline and dopamine rich environment. However, neurons containing phenylethanolamine *N*-methyltransferase, an enzyme required for the synthesis of adrenaline from noradrenaline, are lacking in the intestinal mucosa [16, 17], making it unlikely that adrenaline would normally be found in significant quantities in the gut, except perhaps when the mucosal barrier has undergone damage [18].

Table 1 reveals that prokaryote responsiveness (in terms of enhancement of growth/virulence) to eukaryotic catecholamine hormones is widespread. What is most noticeable is that the spectrum of stress hormone responsive microbes is weighted towards bacteria inhabiting the gastrointestinal tract, particularly species such as *E. coli*, *Salmonella*, *Helicobacter*,* Listeria, Campylobacter*, and *Yersinia* [13, 14, 18–43]. Adrenaline is not produced within the ENS [16, 17], which correlates well with the results of a set of comparative analyses of catecholamine growth responsiveness of three enteric pathogens characterized by their tendency to primarily inhabit the gut (*Y. enterocolitica*) or to colonize extraintestinal sites (*E. coli* O157:H7 and *S. enterica*). This study [40] found a distinct preference of all the bacteria for the gut catecholamines noradrenaline and dopamine over adrenaline. In the case of *Y. enterocolitica*, there was no growth responsiveness to adrenaline, and the adrenergic catecholamine actually competitively blocked *Y. enterocolitica* responses to noradrenaline and dopamine. These results suggest that bacteria have evolved catecholamine response systems specific for the hormone they will encounter within their particular host niche.

Catecholamines are physiologically ubiquitous in terms of their signalling functions and they are utilised in organs and tissues throughout the mammalian body [15]. Thus, it might be expected that bacteria occupying a variety of *in vivo* niches will at some point come into contact with catecholamines and so have cause to evolve sensory systems for monitoring the stress hormone levels of their host. The fact that microbes inhabiting other regions of the body such as the lungs or skin are catecholamine responsive (Table 1) seems to support this hypothesis. In serum-based media, several log-fold increases in cell numbers of *Pseudomonas aeruginosa *[13, 14, 44, 45],* Klebsiella pneumoniae*, [14], *Bordetella pertussis*, and *B. bronchiseptica* [46, 47] have been reported. Although no effects on growth were observed, O'Neal et al. used microarrays to show that noradrenaline upregulated expression of genes required for host tissue attachment in *Mycoplasma hyopneumoniae* [48]. Stress is a well-recognised risk factor for the development of periodontal disease, an oral health problem that accounts for more human tooth loss than dental caries. Oral bacteria are implicated in causing periodontitis, whose incidence is also increased when patients are stressed. A connecting observation may be the study of Roberts et al. [49, 50] who found that those periodontal pathogens most recognised as being causative agents of gum disease displayed the greatest stress hormone responsiveness.

Skin-associated bacteria, particularly the coagulase-negative staphylococci, are very stress hormone responsive [14, 51–53]. Noradrenaline, adrenaline, dopamine, and the synthetic catecholamine inotropes dobutamine and isoprenaline, were all able to increase staphylococcal growth in blood or serum based media by up to 100,000-fold over controls [14, 52]. Formation of a biofilm is a particularly important aspect of bacterial pathogenesis as it enables the microbe to avoid the attack from therapeutic antimicrobials and the host's immune defences [53]. The coagulase-negative staphylococci are considered to be of low pathogenicity but can pose a significant infection challenge for acutely ill patients because of their ability to colonise and form biofilms within intravenous (IV) lines. Catecholamines at the levels infused down IV lines were found to enhance staphylococcal biofilm formation on the same plastic polymers used in line manufacture [53]. Clinical levels of catecholamines also stimulated *P. aeruginosa* biofilm formation on endotracheal tubing (used to maintain an open airway in ventilated patients) [45].

## 4. Noncatecholamine Microbial-Hormone Interactions

Evidence also exists that a variety of noncatecholamine mammalian hormones are recognised by pathogenic microorganisms. For example, *Burkholderia pseudomallei* has a specific and high affinity binding site for insulin [54], which might explain why in human patients with diabetes the progression of melioidosis has been shown to be influenced by serum insulin levels. A binding activity for thyrotropin (a hormone of the anterior pituitary gland which stimulates the thyroid gland) has been isolated from *Y. enterocolitica* [55]. Use of radiolabelled thyrotropin showed that the thyrotropin specificity of the *Y. enterocolitica* binding activity was similar to that of the thyrotropin receptor in human thyroid tissue. Opioids are an effector released by certain mammalian tissues under stress including those within the gut, which makes interesting the finding that *P. aeruginosa* responds to the opioid dynorphin with significantly increased infectivity [56]. A number of other studies have for some years demonstrated the importance of female reproductive hormones on the pathogenicity of yeast involved in urogenital infections [57–61]. *Candida albicans *has been shown to bind human chorionic gonadotropin and interact with human luteinizing hormone which was found to stimulate yeast adenylate cyclase activity and germ tube formation [58–60]. Oestrogen has also been shown to enhance *C. albicans *infectivity, inducing the morphological switch from yeast to the more invasive hyphal form [61] which may explain why there is an increase in the susceptibility of pregnant women to development of *Candida* infections.

## 5. Molecular Studies of Bacterial-Hormone Interactions

Studies into the effects of catecholamine stress hormones on bacterial infectivity have fallen into 3 principle areas: growth, virulence, and gene expression [7]. Most stress hormone-bacteria interaction studies have typically used levels of catecholamines in the region 50 *μ*M–5 mM [2–4, 7] and have largely concentrated on bacterial interactions with noradrenaline and adrenaline (Table 1); however, one human-focussed study using *P. aeruginosa *employed the levels of catecholamines found within the circulation of intensive care patients [45].

### 5.1. Mechanisms of Catecholamine Growth Induction

Most analyses of bacterial stress hormone responsiveness have been conducted *in vitro* and have employed serum- or blood-based culture media to more closely reflect the challenging nature of the host environment in which the microbe will encounter the hormone [4]. Blood or serum containing media are bacteriostatic through the sequestration of free Fe by high affinity ferric iron binding proteins such as transferrin and lactoferrin [62, 63]. Because iron is so essential for the *in vivo* proliferation of bacterial pathogens [62], its limitation by transferrin and lactoferrin is a key innate immune defence against infection [63]. In terms of how catecholamine stress hormones induce growth, it has been shown that catecholamines can act as a kind of siderophore [64–66] which enables bacteria to access the normally unavailable Fe within transferrin and lactoferrin [23, 24, 40, 47, 51–53, 64, 65]. Mechanistically, adrenaline, noradrenaline, and dopamine have been shown to form a complex with the ferric Fe present within transferrin and lactoferrin [64]. The use of electron paramagnetic resonance spectroscopy and biochemical analyses showed that catecholamines reduce the ferric iron to ferrous, a valency for which the iron binding proteins have a much lower affinity [63]. The Fe(III) to Fe(II) reduction weakens the bond between the iron and transferrin and lactoferrin, causing Fe release which can then be taken up by bacteria [23, 24, 35, 40, 43, 45, 47, 51–53, 64, 65]. This bacterial hijacking of host hormones to steal normally secure host Fe is highly relevant to the infectious disease process, as in less than 24 hours the growth enhancement in serum or blood resulting from the addition of catecholamines can be >100,000-fold over control cultures [4, 14, 23, 24, 51–53].

Certain of the metabolites of noradrenaline, adrenaline and dopamine still in possession of the catechol moiety (such as dihydroxy mandelic acid and dihydroxyphenyl glycol) (Figure 1) can also induce bacterial growth to the same level as the parent molecule [24, 52] even though such metabolites are considered to be pharmacologically inactive [15]. Tyramine, which is structurally related to dopamine and abundant in several dairy products owing to the tyrosine decarboxylase activity of *Enterococcus faecalis* starter cultures, was found to increase adherence of *E. coli* O157:H7 to murine caecal explants although it did not stimulate growth [30]. In addition, extracts of banana that are rich in noradrenaline and serotonin [67] can also enhance growth of Gram-negative bacteria [68]. In addition to catecholamine hormones and their metabolites [23, 51], plant extracts containing catechol groups (such as catechin, caffeic, chlorogenic, and tannic acids) have the ability to stimulate growth in serum or blood growth also by enabling bacteria to acquire iron from transferrin and lactoferrin [35].

It has been shown for Gram negative bacteria such as *E. coli* that siderophore synthesis and ferric iron transport systems are required for catecholamine growth induction. Strains mutated for enterobactin synthesis (entA or entF) or ferric-enterobactin transport (*cir, IroN*, *or tonB*) were not able to respond with increased growth to catecholamines in iron-limited serum [65, 66]. It is thought that the siderophore binds and internalises the ferric iron removed by the catecholamine. However, as already mentioned since the reduction of transferrin and lactoferrin Fe(III) by the catecholamine may also occur, incorporation of released Fe(II) by bacterial ferrous uptake systems is also possible [64].

Another mechanism by which catecholamines can induce growth of Gram-negatives involves the production of a bacterial stimulator of growth [14, 20]. This growth stimulator was termed the noradrenaline-induced autoinducer (NE-AI) because it induces its own synthesis and also to distinguish it from the homoserine lactone AIs involved in quorum sensing. The NE-AI is principally produced by Gram-negative enteric bacteria; it is heat stable and has broad cross-species functionality, inducing increases in growth in blood or serum to a level similar to that obtained with the catecholamines [14]. The mechanism by which the NE-AI stimulates growth is unclear but is independent of transferrin or lactoferrin [65]. The NE-AI may have a role in bacterial pathogenicity as it was found to revive viable but nonculturable *E. coli* and *Salmonella* [69]. In terms of induction of the NE-AI production, Lyte et al. showed that only a single 4–6 hour exposure to the catecholamines is needed [20] after which the activity induces its own synthesis. This suggests that enteric bacteria are able to retain a “memory” of even a transient encounter with their host's stress hormones and that catecholamine release during a short-term acute stress could have lasting and widely acting effects on different species of the gut microflora even after stress hormone levels in their host have returned to normal.

### 5.2. Stress Hormone Effects on Microbial Virulence

In addition to their enhancement of growth in host-like serum- or blood-containing media there is now considerable evidence to suggest that catecholamines can also have direct effects upon the bacterial phenotype and, in particular, modulate expression of genes required for virulence. Most studies have involved enteric pathogens. Noradrenaline has been shown to increase production of Shiga toxins by *E. coli *O157:H7 [21] which may be significant in the clinical context as Shiga toxin can cause acute renal and neurological complications [70]. A number of *in vitro* reports have shown that stress hormones can markedly enhance bacterial attachment to host tissues [25, 26, 28, 29, 31–33, 71]. Vlisidou et al. [31] used a bovine ligated ileal loop model of infection to show that noradrenaline increased the intestinal mucosa adherence and enteropathogenicity of *E. coli* O157:H7. Related studies by Green et al. [28, 29] and Chen et al. [25, 32] also showed that catecholamines can increase attachment of enteric pathogens to gut tissues. Research by Bansal et al. showed that *E. coli* O157:H7 displayed a positive chemotactic response towards noradrenaline and adrenaline [33]. Toscano et al. [39] used a pig model of infection to show that pretreatment of *S. typhimurium* to noradrenaline altered the tissue colonisation by the bacteria. Other workers have shown that chicks directly given noradrenaline by crop instillation had increased numbers of* S. enterica* serovar enteritidis in the caeca and liver compared to controls [41]. Noradrenaline has been shown to modulate the expression of the outer surface protein OspA of *Borrelia burgdorferi* [72] and to alter transcription of the catechol siderophore receptor BfeA of *Bordetella bronchiseptica* [46]. Although most stress hormone-virulence studies have concentrated on aerobic pathogens, recently it was shown that catecholamines can also enhance the virulence of anaerobes. *Brachyspira pilosicoli* is an anaerobic spirochaete that colonizes the large intestine of birds and mammals including occasionally humans; Naresh and Hampson (2011) [71] reported that treatment with noradrenaline enhanced *B. pilosicoli* growth, increased its attraction to mucin, and enhanced host cell attachment.

During stress, as well as catecholamines (which are released by the sympathetic nervous system) the hypothalamic-pituitary adrenal axis also induces glucocorticoid stress hormone release by the adrenal gland [9]. It is therefore interesting that exposure to adrenocorticotropic hormone increased attachment of *E. coli* O157:H7 to colonic mucosa [73]. A study by Verbrugghe et al. [74] showed pig social stress and starvation result in elevated serum cortisol levels and that cortisol increased intracellular proliferation of *Salmonella* in primary porcine alveolar macrophages.

### 5.3. Mammalian Hormone Effects on Microbial Gene Expression

In an attempt to profile the global response of bacteria to catecholamine exposure, a number of microarray studies in the presence of adrenaline and noradrenaline have been undertaken. However, in making these transcription profiles there have been considerable variations in the methodologies used (host-like serum-supplemented culture media, nonhost-like laboratory culture media, different catecholamine concentrations, exposure times, and so forth) and so direct comparisons between these studies are not straight forward (see [75] for a fuller consideration). Considered as a collective, the gene expression profiles of catecholamine-treated bacterial pathogens generally support the *in vitro* and *in vivo* studies and the view that exposure to catecholamine stress hormones enhances expression of genes involved in bacterial pathogenicity (such as motility, toxin production, iron acquisition, and host cell attachment) (e.g., [34, 48, 76, 77]). However, a microarray analysis of the effects of the noncatecholamine stress hormone cortisol on *Salmonella typhimurium* gene expression in a laboratory culture medium (Luria broth or Dulbecco's Modified Eagle Medium, DMEM) did not reveal any effects on virulence gene expression [74].

In terms of microbial interactions with noncatecholamines, most studies have focused on fungal interactions with human steroidal sex hormones. For instance, Banerjee et al. (2007) [78] carried out microarray analysis of *C. albicans* treated with progesterone. A total of 99 genes were differentially regulated by progesterone. It was found that progesterone enhanced the expression of multidrug resistance genes, as well as genes involved in hyphal induction and pathogenesis. Downregulated genes included those involved in subcellular localization, metabolism, protein synthesis, cellular transport, transcription, cell cycle, and DNA processing.

## 6. Evolution of Bacterial Catecholamine Responsiveness 

Why should bacteria have evolved the ability to recognise catecholamines? An answer may be related to the widespread utilisation of catecholamines as signalling molecules in nature and the need for microbes to find a suitable host. The evolution of microorganisms preceded that of multicellular life, and it has been demonstrated that catecholamines are widely dispersed throughout the animal and plant kingdoms. In plants, catecholamines are major signaling molecules in many species where they direct processes such as fertilization and fruit and seed development [79]. Dopamine, serotonin, and noradrenaline have been obtained from bananas [67], and the L-dopa (precursor of dopamine) level of broad beans is so high that consumption of 250 g of cooked beans daily has been used to treat the symptoms of Parkinson's disease in human patients [80, 81]. Dopamine has also been found in several species of fungi [82]. It is important to appreciate that the catecholamines isolated from plants and fungi are not analogues of the vertebrate hormones but are chemically identical. This ubiquitous utilisation of catecholamines throughout the eukaryotic world suggests that during evolutionary time microorganisms would have had ample opportunities to come into contact with catecholamines and to develop sensory systems which recognize them as indicators they are within the locality of a suitable host.

In animals, adrenaline, noradrenaline, and dopamine signal by binding to specific adrenergic, noradrenergic, and dopaminergic receptors; catecholamine receptor binding can be prevented using an antagonist specific to the catecholamine receptor, a strategy which has been utilised in treating human health conditions such as hypertension [15]. Intriguingly, it has also been shown that the antagonists of mammalian adrenergic and dopaminergic receptors can also block catecholamine responses in bacteria [43, 51]. Addition of *α*- (but not *β*-) adrenergic receptor antagonists such as phentolamine and prazosin blocked Gram-negative and Gram-positive pathogen growth responsiveness to noradrenaline and adrenaline, as would occur in animal systems [15]. However, the alpha antagonists did not affect growth stimulation by dopamine, and, conversely, as would occur in animals, the dopaminergic receptor antagonist chlorpromazine blocked bacterial responses to dopamine but not to either adrenaline or noradrenaline [43, 51]. This suggests that bacterial response systems exist for catecholamine recognition that possess a degree of specificity similar to that demonstrated for mammalian catecholamine receptors.

In terms of the identification of a specific bacterial catecholamine receptor, there is so far no genomic evidence for the existence of a classical adrenergic or dopaminergic receptor motif in bacterial species. However, Clarke et al. [83] used *in vitro* constructs to show that noradrenaline and adrenaline could bind to the *E. coli *O157:H7 two-component regulator sensor kinase QseC, leading to the proposal that this was a bacterial receptor for these catecholamines. In addition to adrenaline and noradrenaline QseC was found to also recognise a microbial signal (termed AI-3) whose production is indirectly associated with the LuxS AI-2 quorum sensing pathway. This apparent cross-over in signal recognition led to the suggestion that there is a connection between *E. coli* intrakingdom (quorum sensing) and interkingdom (microbial endocrinology) signalling pathways [83]. The *Salmonella* QseC has also been proposed to be a catecholamine receptor and important for virulence, though there are contrasting reports regarding this role [84–86]. Mutation of the *Salmonella* QseC [86] did not as would be expected block bacterial responsiveness to adrenaline or noradrenaline. In the case of infection of calves, inactivation of QseC also did not appear to affect *Salmonella *virulence as no difference was observed in intestinal colonisation from wild type [86]. Noradrenaline and adrenaline are both catechol-containing compounds, which makes interesting the recent report from Karavolos et al. [85] that the catechol-containing 2,3-dihydroxybenzoylserine could also activate an AI-3 reporter. In addition, Haigh et al. [87] found that LuxS inactivation in pathogenic *E. coli* did not affect the ability of the quorum sensing mutants to respond to adrenaline, noradrenaline or dopamine. These studies suggest that response system(s) for the recognition of catecholamines exist in enteric bacteria that are additional to QseC and which do not require factor(s) whose synthesis is dependent on LuxS [85–87].

There is also now some evidence that catecholamines may even shape the evolution of enteric bacteria. Peterson et al. showed *in vitro* that noradrenaline increased the horizontal gene transfer efficiencies of a conjugative plasmid from a clinical host strain of *S. typhimurium* to an *E. coli* recipient [88]. This suggests that the stress of the host could be an additional factor that influences the evolution and adaptation of their microflora.

## 7. Stress, Farm Animal Health and Food Safety

### 7.1. Animal Stress and Infection

Stress is a term used to describe experiences that are challenging psychologically or physiologically; a stressor is the stimulus that causes the stress and can be physical, psychological, or both. In animals stress results in a bidirectional communication between the brain and the peripheral organs and is mediated by a variety of hormones and neuroactive factors [9]. This communication is so intricate that stressful stimuli perceived by the central nervous system (CNS) can directly affect organ functioning, and physiological changes within the organs of the body can directly affect the CNS. Nearly all immune cell classes possess receptors for stress-related hormones adrenaline and noradrenaline, and sympathetic nerve fibres extensively innervate lymphatic tissue, such as bone marrow, thymus, spleen, and lymph nodes, and terminate in close proximity to lymphocytes [9]. In the context of infection, perception of stress by the CNS leads to release of stress associated chemicals which can directly affect immune function, usually resulting in impairment with implications for a variety of health conditions, particularly infection [9, 89].

A number of animal studies have shown that psychological and physical stress can affect the microflora of an animal. Psychological stress can directly affect the behaviour of bacteria present *in vivo* as Dréau et al. showed that the growth of pathogenic *E*.* coli* present in semipermeable chambers implanted within the peritoneal cavity of mice was significantly increased when the animals had experienced a social conflict stress [90]. The implanted chamber was open to protein and hormonal factors within the animal's system but not to cells, suggesting that the bacteria were responding to soluble factors associated with the host stress. Intestinal overgrowth of commensal *E. coli*, which can result in serious systemic infection, has been shown to occur in mice that had been exposed to psychological stress such as restraint [91]. Belay and Woart found that cold stressing mice resulted in elevated plasma noradrenaline and adrenaline levels and increased the susceptibility of the stressed mice to *Chlamydia trachomatis* infection [92]. Bailey et al. [91] also found that psychologically stressing mice altered their microbial gut diversity to such an extent that it increased the capacity for an invading enteric pathogen (*C. rodentium*) to establish an infection. Spillover of catecholamines from the systemic circulation into the gut has been shown to occur during acute stress, and increased release of catecholamines by the gut nerves during stress has also been demonstrated experimentally [93, 94]. In rats, intestinal expression of tyrosine hydroxylase (involved in catecholamine biosynthesis [15]) becomes upregulated in response to surgical injury to the bowel and gut-derived sepsis [95]. Physically stressing mice by surgery (in the form of a partial hepatectomy) or via a short-term period of starvation was found to significantly increase the numbers of *E. coli* adhering to the caecal mucosa of the stressed mice compared to control animals [44]. Collectively, these studies show that the psychological and physical stress of a host is somehow being sensed by its microflora, and, in the case of pathogens, the host stress is apparently being answered with increased virulence.

### 7.2. Stress and Farm Animal Welfare and Productivity

Farm animals are highly sensitive to their living environments, which when changed can be a major source of stress [96]. Cows and cattle are amongst the most studied in this context, and several reports have shown that changes in environmental temperatures, restraint, isolation from herd members, negative social experiences, and physical stress resulting from inadequate food or fluid intake can all have significant impact on the well-being of farmed animals [96]. Many types of stressors can reduce livestock productivity [97, 98]. It has been shown for dairy cows that psychological stress in the form of shouting or prodding can significantly reduce milk output [97]. Handler-related stress was found to increase release of the stress hormone cortisol into the cow's milk, and the stressed animals also retained a memory of the negative treatment and later showed a higher incidence of stressed avoidance behaviour towards handlers. In contrast, animals treated gently subsequently showed less fear towards humans and were easier to handle [96]. Another study [99] found that cows stressed by human handlers exhibited negative changes in feed intake and rumen function. Discomfort or pain can affect the behaviour of all animals and is itself a major stressor. Stress in livestock has been shown to activate the adrenocortical axis, leading to a reduction in the animal's tolerance to pain [98]. Significantly, the stressors inducing this nociception included procedures routinely used in farm animal management such as social isolation in novel surroundings, head fixation by tethering, and introduction of unfamiliar neighbours into adjacent stalls.

Increased temperature is a major livestock stressor, negatively affecting the physiology, hormonal balances and growth of cows, cattle, and poultry [100]. The effect of heat stress in cattle has been the most investigated, as overheating is a difficult stressor to control. Tajima et al. (2007) found that the diversity of the rumen microflora in Holstein heifers altered in response to increasing housing temperature [101]. In the heat stressed animals a reduction in volatile fatty acids, which are an indicator of rumen functionality and a major ruminant energy source, significantly reduced heifer weight gain. Later work by Uyeno et al.  (2010) [102] identified the heat stress responsive rumen species as the genus *Streptococcus* and members of the *Clostridium coccoides-Eubacterium rectale* family of bacteria, both of which increased in numbers, and the genus *Fibrobacter*, whose population sizes decreased. The host-associated factors that triggered the changes in microflora diversity were unclear, but it is the case that the rumen microbes were responsive to the physiological changes experienced by their host during the heat stress.

Stress in farm animals may also have implications for the microbiological safety of human meat products, as in a number of studies stress has been shown to correlate with increased excretion of food borne pathogens such as *E. coli* O157:H7 and *S. enterica*. In piglets, stress in the form of isolation from the sow, cold stress, or mixing piglets with those from foreign litters increased faecal excretion of enterotoxigenic *E*.* coli* relative to unstressed piglets [103]. Even a brief human handling of piglets involving weighing was enough to significantly increase faecal excretion of *E. coli *relative to control pigs [104]. Transportation is a routine aspect of life for farm animals, which is of significance as pigs subject to regular transport exhibited increased faecal excretion of the food borne pathogen *S. enterica *[105]. How is the stress of the host linked to changes in the behaviour of the livestock gut microflora? Stress via the sympathetic nervous system can affect gut function, which could in turn affect microbial composition. Restraint stress has been shown to affect secretion of gastric acid and reduce gastric motility [106], which could by changing local physical parameters affect the resident gut microbes. The microbial endocrinology explanation would be that the gut microbes are directly sensing and responding to the stress experienced by their host.

Insight into the molecular mechanisms that may be at play during host stress is suggested by a study by Lyte and Bailey [107] who used a mouse model of acute physical stress involving the selective neurotoxin 6-hydroxydopamine (6-OHDA). The 6-OHDA selectively ablates the nerve terminals of sympathetic neurons and causes the rapid release of stored noradrenaline into the systemic circulation, including the gut, and so mimics the hormonal changes that take place during acute stress. Lyte and Bailey found that the numbers and diversity of bacteria in the caeca of the stressed mice increased by up to 4 logs during the 24 hours following administration of the neurotoxin, with *E. coli* showing the greatest increase. Attachment of the gut bacteria to the mouse caecal wall and translocation to the mesenteric lymph nodes (potentially the beginning of a gut-associated systemic infection) were similarly increased in the stressed mice. Within two weeks, the time typically required for regeneration of the affected nerves, the previously high bacterial counts in the gut had returned to normal [107]. A later *Salmonella *study by Pullinger et al. [108] showed that 6-OHDA treatment of pigs following oral inoculation with *S. enterica *increased plasma noradrenaline levels and enhanced faecal excretion of the pathogen. Oral administration of noradrenaline to the *Salmonella*-infected pigs also increased shedding of the bacteria. A possible connecting link between increased host levels of stress hormones and changes in the commensal microflora is suggested by the finding that growth of commensal intestinal *E. coli* isolates increased by nearly 5 logs following exposure to the gut catecholamines noradrenaline, dopamine and their metabolites [24]. The co-localisation in the gastrointestinal tract of bacteria, lactoferrin and transferrin may therefore explain why large increases in noradrenaline levels that occur during acute stress can catalyse the overgrowth and translocation of the gut microflora [42, 107]. It is therefore not surprising that mammals appear to have evolved protective mechanisms to tightly regulate levels of gut catecholamines and that catecholamine-degrading enzymes are present throughout the entire length of the human gastrointestinal tract [109]. Distribution of the phenol sulfotransferase family of catechol-inactivating enzymes shows a close correlation with the presence of bacteria, with enzyme expression being the lowest in the stomach and the highest in the intestine and colon.

## 8. Catecholamine Inotropes and Human Health

As well as their normal neuroendocrine signalling functions, catecholamines are employed therapeutically as inotropic agents to maintain renal and cardiac function in acutely ill human patients. For example, dobutamine is used to treat congestive heart failure, while adrenaline is used for management of anaphylactic shock and dopamine to support renal function [15]. Surveys of drug usage within ICUs indicate that patients may receive up to 20 medications during their stay, with up to half receiving catecholamine inotropes [110]. Catecholamine levels within the human body are normally tightly regulated and plasma clearance is usually rapid in the healthy human, resulting in circulatory levels in the nanomolar range [15]. However, catecholamine concentrations in patients receiving inotropic support can be much higher. For example, dopamine is typically infused intravenously into acutely ill adult patients over a concentration range of 1–15 *μ*g/kg/min (http://www.bnf.org/) and has a plasma half-life of several minutes [15]. Steady state levels vary according to infusion rates and metabolic fitness, with acutely ill patients typically showing slower elimination rates [111]. There is therefore considerable variation in dopamine plasma concentrations in patients receiving inotrope supplementation and levels can rise to as high as 5 *μ*M [111]. In humans dopamine is metabolized to noradrenaline and adrenaline after infusion and Thompson et al. found that in some cardiac surgery patients treated with 3 *μ*g/kg/min dopamine, noradrenaline plasma levels rose to as high as 9.24 *μ*M [112]. The Thompson and Girbes studies are of particular interest to the field of microbial endocrinology as the catecholamine levels reported are high enough to affect transferrin binding of iron and to enable bacterial growth in blood or serum [4, 45].

Patient-associated risk factors for the development of nosocomial (hospital acquired) infections are thought to come from lowered physical fitness due to prior illness, accidental or intentional tissue trauma such as surgery, immunodeficiency, or colonization by infectious microbes. Other factors recognized as leading to increased risk of infection involve invasive treatment procedures, particularly use of endotracheal tubes (with mechanical ventilation), urinary catheters, surgical drains, and IV catheter lines. An additional risk factor is now recognized as coming from the inotrope medications patients are given [113]. This view came about because catecholamine inotropes have been shown to be involved in staphylococcal colonization of indwelling medical devices such as IV lines due to their ability to stimulate staphylococcal growth and biofilm formation [14, 51–53]. IV catheter-related bloodstream infections are often associated with increases in length of time in intensive care which results in increased hospital costs, and a strategy to combat bacterial line colonization has involved use of antibiotic impregnated catheter polymers such as those containing rifampin or minocycline. However, it was found that either dopamine or noradrenaline enabled staphylococci damaged by either antibiotic to recover to active growth in less than a day, even bacteria that appeared to have been killed by the antimicrobial [51].

Ventilated patients on intensive care are at particular risk of acquiring an infection, particularly if they are receiving mechanical ventilation [114] and ventilator associated pneumonia (VAP) is a complication associated with significant morbidity and mortality. A recent study in the journal *Chest* investigated the role of catecholamine inotropes in the development of VAP by the opportunistic pathogen *P. aeruginosa* [45]. This investigation is particularly noteworthy as it was the first to use concentrations of inotropes equal to or less than those found within the blood of inotrope medicated patients. Using *in vitro* growth and virulence assays and an *ex vivo* model of infection using human respiratory epithelium 5 *μ*M dopamine or noradrenaline were found to markedly increase *P. aeruginosa* growth via induction of synthesis of the siderophore pyoverdine and provision of Fe from serum transferrin. Aspects of virulence such as motility and biofilm formation on endotracheal tubing (sections taken from the tube used to ventilate patients) were enhanced by the inotropes. Clinical levels of dopamine and noradrenaline also facilitated the recovery of *P. aeruginosa* from tobramycin antibiotic challenge. Interestingly, the alternative noncatecholamine inotropes vasopressin and phenylephrine had no effects on the growth and virulence of *P. aeruginosa* [45].

The *P. aeruginosa*-inotrope study [45] suggests that administration of inotropes to patients in intensive care, particularly if high doses are given systemically or via direct local application, may be an unappreciated risk factor in the development of VAP. But does this have implications for other lung infectious microbes? Lung inotrope metabolism systems are highly effective and have been proposed to be alternate sites for direct inotrope administration [115]. Adrenaline (as a 300 *μ*M solution) is also occasionally directly nebulised to reduce airway inflammation [116]. In addition to prescribed inotropes, ventilated patients have been found to be both chronically and acutely stressed, as significant endogenous increases in plasma noradrenaline and adrenaline have been associated with procedures such as endotracheal tube suctioning [117]. Thus there are considerable opportunities for resident bacteria located within the airway or on a ventilator tube to come into contact with both endogenous and administered catecholamines, which may have implications for development of lung infections in the acutely ill patient. This idea has support from Marks et al. (2013) [118] who recently used a tissue culture biofilm model of infection combined with animal infection models to show that treatment of *Streptococcus pneumoniae* biofilms with physiological levels of noradrenaline induced bacteria to disperse from a biofilm. This significantly promoted their colonization of normally sterile host tissues, leading to a much greater spread of pneumococcal infection compared to controls.

## 9. Quorum Sensing: Bacteria-Host Communication

Overcoming host defences is essential for a pathogen to establish an infection *in vivo*, and so they produce an arsenal of protein virulence factors (VFs) to enable them to colonise their host such as toxins, adhesins, secreted degradative enzymes, and exopolysaccharide (biofilms). A challenge for the pathogen is that VFs can be highly immunogenic, so timing of their production is important to avoid premature immune system detection and early eradication by the host. By coordinating VF gene expression to high population density, a pathogen has a greater chance of resisting the host defences through weight of numbers [119]. Bacteria “sense” whether they have reached a suitable population density through the accumulation and recognition of small, diffusible pheromone-like chemical signals released into the surrounding medium during growth. Bacteria perceive that the population has reached a “critical” size (is quorate) and so can more safely activate gene expression when the external levels of the signal reach the required threshold. This linking of gene expression to population density has been termed quorum sensing (QS) [119, 120].

Quorum sensing involves the production of and response to signal molecules termed autoinducers (They are called so because they induce their own synthesis) [119]; this area of research is also referred to as intra-kingdom or intercellular signalling. In terms of their involvement in regulation of virulence, QS communication processes have focused on two main chemical languages. For Gram-negatives, this is the N-acyl homoserine lactones (AHLs) synthesised from methionine [119]. In the case of Gram-positive species, the communication molecules consist of autoinducing peptides (AIPs), which are short peptides ribosomally synthesised and in some cases posttranslationally modified [120]. For both types of QS signal, there exists a wide range of structural variation, indicating that individual species are essentially producing their own specific “language.”

Genes for the Gram-negative QS systems were named after *Vibrio fischeri *luciferase genes, *lux*, the first species in which the phenomenon was recognised; *luxI* encodes the N-acyl-homoserine lactone synthase, while *luxR* encodes the luciferase gene response activator [119, 121, 122]. The s-adenosylmethionine metabolic pathway that leads to AHL synthesis also produces a second autoinducer, termed AI-2 [122]. This autoinducer is produced by many Gram-negative and Gram-positive bacteria and in all instances requires a protein called LuxS. Unlike the species specific AHL and AIP, AI-2 is chemically identical in all AI-2-producing bacteria, which has led to the proposal that AI-2 is a universal QS signal that functions in bacterial interspecies communication; the structure of *V. harveyi* AI-2 was the first to be determined and was shown to be a furanosyl borate diester [119, 122]. Investigation of the role of LuxS in relation to the virulence of bacterial pathogens has therefore been a focus of interest for nearly 20 years. However, because disruption of the activated methyl cycle will also affect general fitness, which may then in turn modulate the virulence of bacteria, a debate had evolved as to the true role of LuxS and AI-2 in intercellular communication and metabolism [123–126]. Therefore, this paper will concentrate on the infection significance of AHL-host interactions.

*P. aeruginosa* is a major opportunistic pathogen, with one of the most intensively studied QS systems. Much of the virulence of this microbe is regulated by its two AHLs, N-3-oxododecanoyl homoserine lactone (3-oxo-C12HSL) and N-butanoyl homoserine lactone (3-oxo-C4HL), which are encoded by 2 temporally separate *lux*-type QS systems, termed *las* and *rhl* [119]. The *las* system also regulates the *rhl* system, and because of this, therefore also much of *P. aeruginosa* virulence including elastase, rhamnolipid, toxin, morphological attributes such as motility and biofilm formation; in total up to 4% of the *P. aeruginosa* genome is regulated by AHLs [127]. The *P. aeruginosa* AHLs are also produced during infection and have been isolated from the sputum of cystic fibrosis patients colonised with *P. aeruginosa* [128]. Inactivation of the two *P. aeruginosa* QS systems usually results in reduced virulence, as *las* and *rhl* mutants are significantly attenuated in their capacity to cause infection [129, 130].

This paper has so far shown that bacteria recognise our signalling molecules, so the obvious question that follows is whether the chemical signals of prokaryotes are able to convey information to eukaryotes. Because homoserine lactones are produced during infection [128], AHL-immune system interactions have been a particular focus of interest [131–134]. Tateda et al. [131] showed that 3-oxoC12HSL speeded up apoptosis of macrophages and neutrophils, while Telford et al. demonstrated that exposure to the AHL inhibited lymphocyte proliferation and production of tumour necrosis factor and downregulated production of IL-12, all being important elements in host infection defence [132]. Mast cells play an important role in allergic inflammation and management of infectious disease. Li et al. used synthesised 3-oxoC12HL to show that the AHL significantly inhibited mast cell proliferation and rapidly induced apoptosis [133]. A related study by Boontham et al. [134] investigated whether AHLs could influence the pathophysiology of patients with severe sepsis. The 3-oxoC12HL inhibited human T-cell activation and induced apoptosis in dendritic and CD4+ T cells. Protective proinflammatory cytokine expression was also decreased in the presence of the 3-oxoC12HL. Importantly, a correlation was found between AHL presence in the serum of patients and severity of the sepsis and disease outcome [134].

While AHLs can clearly modulate fundamental processes within animal cells, examples also exist within the plant world of how QS signals can modulate host biology to the benefit of the infecting microbe. *Agrobacterium tumefaciens* is a plant pathogen which uses AHL signalling to control conjugative exchange of a plasmid (Ti) containing genes needed for infection of plant tissue [135, 136]. Following infection, under control from the bacteria the plant forms crown gall tumours, which release opines, carbohydrates used by the bacteria as a nutrient source. Interestingly, the tumour opines also activate expression of the *A. tumefaciens traR*, an AHL receptor which normally is only expressed when opines are present. Activation of TraR increases Ti plasmid exchange and enhances the infectivity of the *A. tumefaciens*.

The mammalian cell-AHL interactions results [131–134] suggest that as well as regulating virulence, an AHL could also act as a direct virulence factor, conveying a message to its animal host to turn off processes that would prepare immune defences for action, even tricking some immunoprotective cells into prematurely entering programmed pathways that lead to their death. Such suppressive activities would favour survival of infectious bacteria and increase their potential to proliferate and establish an infection *in vivo*. Because of the importance of QS in regulating bacterial virulence [119, 120], it is not surprising that mechanisms have evolved that interfere with QS, a phenomenon termed quorum quenching. For instance, *Bacillus *species release an enzyme, AiiA, which can inactivate a wide variety of AHLs by cleaving the lactone ring [137]. This destroys the information within the QS signal, disadvantaging the Gram-negative species that produced it but not the Gram-positive *Bacillus* as it signals via an AIP. Mammalian respiratory airway cells also produce AHL inactivating enzymes, in this case a paraoxonase, which is able to degrade the *P. aeruginosa *3-oxoC12HL [138]. Another work has shown the existence of 3 families of paraoxonase enzymes, Pon1, Pon2, and Pon3, which in mammals are mainly located within the liver; all the paraoxonases can inactivate a variety of AHLs [139]. This suggests that during evolutionary time mammals have learned to recognise the AHL as harmful and to develop an innate means of destroying the signal, protecting the host from the damaging false information it carries and disadvantaging the pathogen by disrupting control of its virulence.

## 10. Conclusion

It is now clear that a complex bidirectional communication is taking place between microorganisms and their host and that information exchange via chemical signals has been part of this relationship for a very long time. This paper has concentrated on languages from the hormonal and quorum sensing alphabets. However, a very recent report [145] showed that immune signalling molecules are also recognised by bacteria, suggesting that the lexicon of interkingdom languages is likely to expand. This emphasises that deciphering what is being said between bacteria and their hosts is needed for understanding the evolutionary biology of chemical signal development. Also, in terms of practical applications, greater understanding of the mechanisms mediating the host*⇔*bacteria communication processes could lead to strategies which disrupt the more damaging aspects of the information exchange and in so doing provide new treatments for bacterial infections.

## Figures and Tables

**Table 1:** Stress hormone responsive bacteria.

Species	Catecholamine/metabolite	Growth	Virulence	Reference
*Aeromonas hydrophila *	NE	+	+	[140]
*Acinetobacter lwoffii *	NE	+		[14]
*Bordetella bronchiseptica*,* B. pertussis *	NE, Adr, Dop	+	+	[46, 47]
*Borrelia burgdorferi *	NE		+	[72]
*Brachyspira pilosicoli *	NE	+	+	[71]
*Campylobacter jejuni *	NE	+	+	[141]
*Citrobacter freundii*, *C. rodentium *	NE	+		[14, 91]
*Enterobacter agglomerans, E. sakazakii *	NE	+		[14]
*Enterococcus faecalis*, *E. cloacae *	NE	+		[14]
*Escherichia coli* (commensal and pathogenic)	NE, Adr, Dop, Iso, Dob, DHPG, DHMA	+	+	[13, 14, 19–35]
*Hafnia alvei *	NE	+		[14]
*Helicobacter pylori *	NE	+		[36]
*Klebsiella oxytoca, K. pneumoniae *	NE	+		[14]
*Listeria monocytogenes *	NE, Adr, Dop	+		[14, 37]
*Morganella morganii *	NE	+		[14]
*Mycoplasma hyopneumoniae *	NE		+	[48]
*Proteus mirabilis *	NE	+		[14]
*Pseudomonas aeruginosa *	NE, Adr, Dop	+	+	[13, 14, 44, 45]
*Salmonella enterica*, *Salmonella typhimurium *	NE, Adr, Dop	+	+	[13, 14, 38–42, 45]
*Shigella sonnei*, *S. flexneri *	NE	+		[14, 142]
*Staphylococcus aureus *	NE, Dop	+		[51]
*Staphylococcus epidermidis, S. capitis, S. saprophyticus, S. haemolyticus, S. hominis *	NE, Adr, Dop, Iso, Dob	+	+	[14, 51–53]
*Streptococcus dysgalactiae *	NE	+		[14]
*Vibrio parahaemolyticus, V. mimicus, V. vulnificus *	NE, Adr, Dop	+	+	[76, 143, 144]
*Xanthomonas maltophilia *	NE	+		[14]
*Yersinia enterocolitica *	NE, Adr, Dop,	+		[13, 14, 40, 43]
**Oral bacteria**	NE, Adr	+		[49, 50]
*Actinomyces gerencseriae, *		+		
*A. naeslundii, A. odontolyticus *		+		
*Campylobacter gracilis *		+		
*Capnocytophaga sputigena, C. gingivalis *		+		
*Eikenella corrodens *		+		
*Eubacterium saburreum *		+		
*Fusobacterium periodonticum, *		+		
*F. nucleatum subsp. vincentii *		+		
*Leptotrichia buccalis *		+		
*Neisseria mucosa *		+		
*Peptostreptococcus anaerobius, *		+		
*P. micros, *		+		
*Prevotella denticola, P. melaninogenica *		+		
*Staphylococcus intermedius *		+		
*Streptococcus gordonii, *		+		
*S. constellatus, S. mitis, S. mutans, S. sanguis *		+		

The “+” indicates that catecholamine stress hormones, inotropes, or their metabolites have induced enhancement of growth or virulence of the bacterial species shown. NE: noradrenaline; Adr: adrenaline; Dop: dopamine; Iso: isoprenaline; Dob: dobutamine; DHPG: dihydroxy phenylglycol; DHMA: dihydroxy mandelic acid.

This table was adapted with permission from Freestone et al. (2008) [3].
